# Near infrared spectroscopy (NIRS) data analysis for a rapid and simultaneous prediction of feed nutritive parameters

**DOI:** 10.1016/j.dib.2020.105211

**Published:** 2020-01-31

**Authors:** Samadi, Sitti Wajizah, Agus Arip Munawar

**Affiliations:** a Department of Animal Husbandry, Syiah Kuala University, Banda Aceh, Indonesia; b Department of Agricultural Engineering, Syiah Kuala University, Banda Aceh, Indonesia; c Agricultural Mechanization Research Centre, Syiah Kuala University, Banda Aceh, Indonesia

**Keywords:** NIRS, Feed, Nutritive value, Prediction, Spectroscopy

## Abstract

Presented paper described dataset on near infrared spectroscopy (NIRS) used as a rapid and robust method to predict and determine several nutritive parameters of animal feed simultaneously. Near spectra data were acquired and recorded in wavelength range from 1000 to 2500 nm with co-added of 64 scans per sample measurement. On the other hand, actual reference nutritive parameters: in vitro organic matter digestibility (IVOMD), in vitro dry matter digestibility (IVDMD), neutral detergent fibre (NDF) and acid detergent fibre (ADF) of animal feed were measured using proximate laboratory procedures. Near infrared datasets can be enhanced using several spectra correction methods to improve prediction accuracy and robustness. Animal feed nutritive parameters can be determined simultaneously and rapidly by establishing prediction models by means of principal component regression (PCR), partial least squares regression (PLSR) and other regression approaches.

**Table:** Specifications Table

Subject	Agricultural and Biological Sciences Animal sciences
Specific subject area	Spectroscopy, non-destructive test for animal feed quality evaluation
Type of data	Table Graph Spectroscopic data
How data were acquired	Spectral datasets of animal feed samples were acquired using a benchtop Fourier transform infrared spectroscopy (*Thermo Nicolet Antaris II TM*). Spectra data were recorded in form of absorbance spectrum in wavelength range from 1000 to 2500 nm with co-added of 32 scans with resolution windows of 0.2 nm. On the other hand, to obtain actual reference data of neutral detergent fibre (NDF) and acid detergent fibre (ADF), standard laboratory procedures as proposed by Ref. [1] is employed. Feed samples were boiled in neutral and acid detergent solutions for an hour in sequential. NDF and ADF data were expressed in percent. Meanwhile, in vitro organic matter digestibility (IVOMD) and in vitro dry matter digestibility (IVDMD) were determined by subtracting residues of dry matter and organic matter prior to fermentation, respectively. The in vitro incubation was carried out in three runs and two serum bottles represented for each run. Those four nutritive parameters were performed in triplicate and averaged.
Data format	Raw Analysed Enhanced Presented as *.xls* and *.unsb* file formats
Parameters for data collection	Nutritive parameters of animal feed samples were in vitro organic matter digestibility (IVOMD), in vitro dry matter digestibility (IVDMD), neutral detergent fibre (NDF) and acid detergent fibre (ADF).
Description of data collection	Near infrared spectroscopic data in form of absorbance spectrum were used to predict four mentioned animal feed nutritive parameters (IVOMD, IVDMD, NDF and ADF) simultaneously. Prediction data of these nutritive attributes were obtained by establishing models through calibration. Principal component regression (PCR) and partial least square regression (PLSR) were applied as methods in collecting prediction values. Prediction data were then quantified by means of cross validation during calibration phase.
Data source location	Data were collected at the Department of Animal Husbandry, Bogor Agricultural University and Department Agricultural Engineering, Syiah Kuala University, Banda Aceh – Indonesia.
Data accessibility	Dataset are available on this article and can be found in Mendeley repository data: https://data.mendeley.com/datasets/r6fjtx943d/1 or https://doi.org/10.17632/r6fjtx943d.1

**Table:** 

**Value of the Data** • Spectral dataset of animal feed samples can be used to predict nutritive parameters derived from calibration models. It provides a rapid, non-destructive and simultaneous approach to determine nutritive attributes of biological objects like animal feed in this case. • Data were benefited in animal feed industries for quality inspection of their feed products. This dataset can also be re-used to develop prediction models for other nutritive parameters like starch, protein, pH and others. • Spectral dataset can be enhanced using several data pre-processing approaches and transferred onto established NIRS instrument. • Prediction performances may vary, depends on spectra enhancement and regression approaches to be applied during calibration and prediction models development.

## 1 Data

**Fig. 1:**
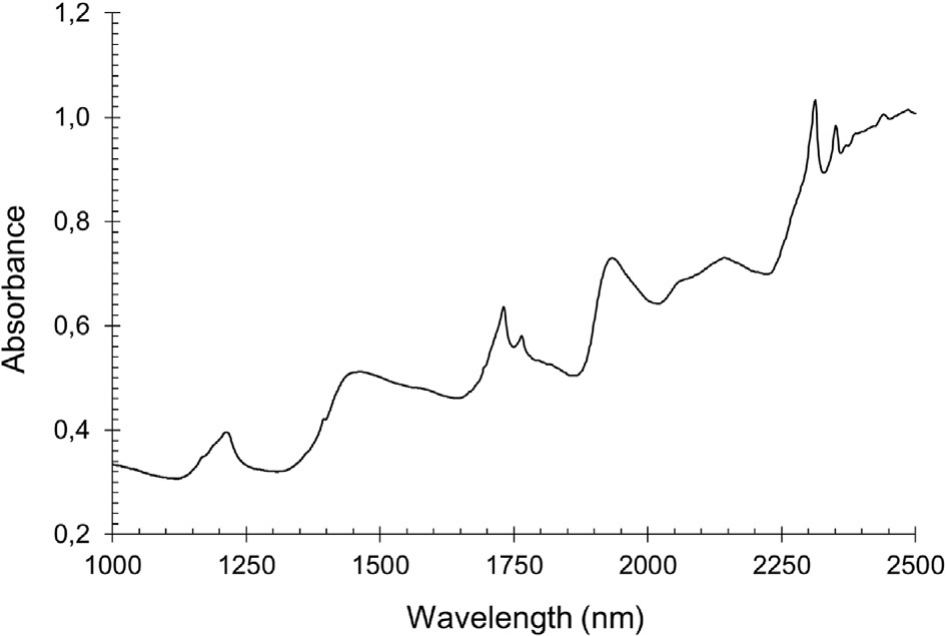
Typical near infrared absorbance spectrum of feed sample.

Near infrared spectral dataset of feed samples were acquired and recorded as absorbance spectrum in wavelength range from 1000 to 2500 nm (Fig. 1). Typically, the near infrared spectra data can be represented as a function of the energies (cm^−1^) or wavelengths (nm) of the electromagnetic radiation. Spectra data contains chemical properties and information that can be revealed through calibration by means of regression approaches. Beside relevant and important information, spectra data may also contain irrelevant information background known as noises due to light scattering [2]. These noises can interfere prediction accuracy and robustness resulted during calibration. Thus, in order to eliminate or minimize noises, spectra data can be corrected and enhanced using several pre-processing techniques such as spectra smoothing, normalization, multiplicative scatter correction (MSC), standard normal variate (SNV), orthogonal signal correction (OSC), spectra derivatives, de-trending, and combination among them [3]. The selection of spectra pre-processing method must be followed by the knowledge of the sample characteristics, sample measurement protocol, radiation interaction, and the requirements of the analytical problem [4].

**Fig. 2:**
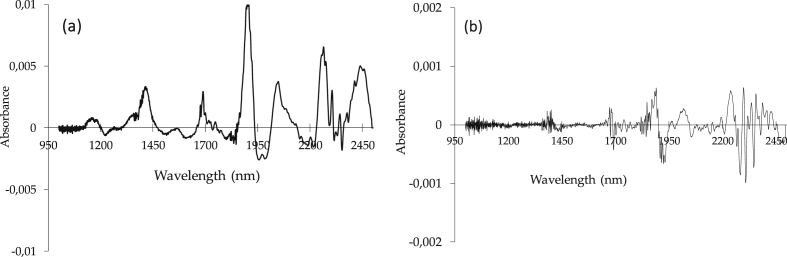
Near infrared spectrum after first derivative (a) and second derivative (b).

Nutritive parameters such as NDF, ADF, pH, starch content, moisture content, IVOMD, IVDMD and others are buried in the spectra pattern along near infrared region. Specific wavelengths are corresponded to certain nutritive attributes from which represents the amount of light absorbed, reflected or transmitted. Light absorption and scattering of the NIR radiation are two main phenomena affecting the featured spectrum [5]. Spectra data can also be transformed into its derivative as presented in Fig. 2 to observe spectra feature more details and detect potential noises along near infrared wavelength region. Spectra derivatives is a good way to correct these distortions which commonly employed a *Savitzky-Golay* derivative algorithm.

The main aim of NIRS application is to establish models used to predict and determine nutritive or quality attributes of studied samples. In last few decades, multiple linear regression (MLR) was firstly applied with a few original variables previously transformed and selected to carry the relevant and important chemical properties information. Nowadays, other regression approaches like principal component regression (PCR) and partial least squares regression (PLSR) are usually employed [6].

**Fig. 3:**
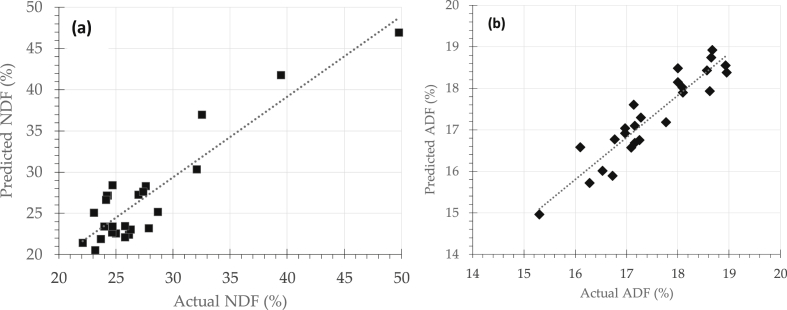
Prediction performance of NDF determination (a) and ADF (b).

These two regression methods were fitted like a glove in the emerging field of NIRS till now. Both methods seek to find best correlation between NIRS spectra data and respective nutritive or quality parameters such as NDF, ADF, IVOMD, and IVDMD in this case. Prediction models were developed by regressing spectra data (X variable) and actual nutritive attributes obtained by standard laboratory procedures (Y variable). Predicted results were then compared to the actual one, in order to judge the prediction performance as shown in Fig. 3.

Prediction models can be developed directly either using raw (un-treated) spectra data or enhanced (treated) spectra [7]. As mentioned previously, it is necessary to correct and pre-process the near infrared spectra data in order to achieve more accurate and robust prediction results. Table 1 showed a comparison of prediction performance between raw and corrected spectra data for feed nutritive parameters determination.

**Table 1:** Comparison of prediction performance between raw and corrected spectra data using principal component regression (PCR) approach.

Nutritive Parameter	Spectra data	Statistical indicators			
		R^2^	r	RMSE	RPD
NDF	Raw	0.757	0.870	3.665	1.627
	Corrected	0.830	0.911	3.098	1.925
ADF	Raw	0.831	0.912	0.538	1.782
	Corrected	0.867	0.931	0.486	1.972

ADF: acid detergent fibre, NDF: neutral detergent fibre, R^2^: coefficient of determination, r: correlation coefficient, RMSE: the root mean square error, RPD: residual predictive deviation.

Prediction performance may vary depends on which spectra pre-processing algorithms and regression approach to be used. As presented in Table 2, prediction performances were varied among different spectra correction methods were in this data analysis using standard normal variate (SNV), baseline shift correction (BSC), and de-trending (DT) generally improved when the models are constructed using corrected spectra data.

**Table 2:** Comparison among different spectra correction methods to the prediction performance of nutritive parameters using partial least squares regression (PLSR) approach.

Nutritive parameters	Spectra data	Statistical indicators			
		R^2^	r	RMSE	RPD
IVOMD	Raw	0.590	0.770	2.510	1.599
	SNV	0.810	0.922	1.710	2.347
	BSC	0.750	0.861	1.960	2.048
	DT	0.690	0.832	2.180	1.841
IVDMD	Raw	0.610	0.780	2.032	2.284
	SNV	0.860	0.936	1.201	3.911
	BSC	0.720	0.861	1.651	3.706
	DT	0.720	0.852	1.712	3.362

BSC: baseline shift correction, DT: de-trending, IVOMD: in vitro organic matter digestibility (IVOMD), IVDMD: in vitro dry matter digestibility, R2: coefficient of determination, r: correlation coefficient, RMSE: the root mean square error, RPD: residual predictive deviation. SNV: standard normal variate.

## 2 Experimental design, materials, and methods

### 2.1 Instrument setup

Near infrared spectra data of feed samples were acquired using a benchtop NIR instrument (Thermo Nicolet Antaris II TM). The instrument was controlled and configured under integrated software Thermo Integration® and Thermo Operation®. Specified tasks were performed by establishing workflow using Thermo Integration software. High resolution measurement with integrating sphere was chosen as a method for spectra acquisition [8].

For each spectra measurement, sample labelling was required automatically prior to acquisition in order to distinct feed samples respectively. Spectra data were acquired and recorded as absorbance spectrum in wavelength range from 1000 to 2500 nm and saved in three different file formats: Nicolet (*.spa*), Jcamp (.jdx) and comma separated value (*.csv*). Standard laboratory methods were also prepared for four mentioned nutritive parameters of feed samples.

### 2.2 Spectra data acquisition

Near spectra data were firstly acquired by means of NIR instrument for all feed samples, made from eight sources of agro-industrial residues by products (sago residues, coconut meal, soybean-ketchup by product, coffee pulp, cacao pod, sago tree, corncob, and rice brand). Around 33 g of a bulk of feed samples were placed centrally upon sample holder. Each bulk sample was hand placed manually right to the incoming hole (1 cm of diameter) of the light source to ensure direct contact and minimize noises due to light scattering. Absorbance spectrum in wavelength range from 1000 to 2500 nm were acquired with co-added of 64 scans. Sample was set to rotate during spectra acquisition to ensure uniformity.

### 2.3 IVOMD, IVDMD, NDF and ADF measurements

Once after spectra data acquisitions were completed, feed samples were directly analysed to determine actual nutritive parameters. For NDF and ADF measurement, feed samples were boiled in neutral detergent and acid detergent solutions for 1 h in sequential. The NDF value was determined without using α-amylase and sodium sulfite, whilst for ADF, both of them were used. Determination of NDF and ADF were based on neutral detergent insoluble CP (NDICP) and acid detergent insoluble CP (ADICP) contents respectively and expressed as exclusive of residual ash [9].

Moreover, in vitro rumen fermentation was performed to determine IVDMD and IVOMD. Briefly, rumen fluid was collected in the morning before feeding through a rumen fistulated cow. Rumen fluid was filtered with four layers of gauze before using [10]. A 125 ml serum bottle was prepared to fill in 0.75 sample and add 75 ml buffered rumen fluid with the ratio of rumen fluid buffer (1.4 v/v). Incubation was taken place in a water bath with the temperature of 39 °C for 48 hours. After incubation, supernatant obtained was analysed to determine pH its residue was further incubated with 75 ml pepsin-HCl 0.2 N solution for another 48 hours [1,10]. Feed nutritive attributes: IVOMD and IVDMD were determined by subtracting residues of organic matter (OM) and dry matter (DM) from initial prior to fermentation respectively. The in vitro incubation was conducted in three replicates and two serum bottles represented for each replicate. Descriptive statistics of actual measured IVOMD, IVDMD, NDF and ADF are shown in Table 3.

**Table 3:** Descriptive statistics of actual measured nutritive parameters of feed samples.

Statistical indicators	Nutritive parameters			
	IVOMD	IVDMD	NDF	ADF
Mean	56.50	54.14	27.36	17.48
Max	64.58	60.36	49.77	18.95
Min	50.34	48.56	22.10	15.30
Range	14.24	11.80	27.67	3.65
Std. Deviation	4.01	3.34	5.96	0.96
Variance	16.11	11.12	35.54	0.92
RMS	56.63	54.24	27.97	17.51
Skewness	0.73	0.40	2.72	-0.20
Kurtosis	-0.34	-0.60	8.34	-0.48
Median	55.85	53.92	25.78	17.25
Q1	54.15	52.09	24.21	16.97
Q3	57.39	55.55	27.61	18.10

ADF: acid detergent fibre, IVOMD: in vitro organic matter digestibility, IVDMD: in vitro dry matter digestibility, NDF: neutral detergent fibre, Q1: first quartile, Q3: third quartile.

### 2.4 Sample outlier detection

In many practices, NIRS users sometimes faced with the problems in selecting the most representative sample datasets for calibration, splitting a large dataset into subsets, aiming at calibration and validation, or identifying and detecting samples that are somehow considerably different from the majority of the remaining samples from which known as outlier(s) [4,11]. These outliers can be found in the sample datasets used for model construction and validation, or arise among new samples during the use of those models for independent prediction.

There are several methods that can be used to detect and remove outliers, one of the most common method is the Hoteling statistics (or t^2^) ellipse, used to define statistical boundaries assuming a normal distribution of scores of principal component analysis (PCA). Typically, outliers are identified as samples found outside or beyond the ellipse confidence limit, usually established at the level of 95% [12,13]. Another recommended method for outlier detection is the use of the *Mahalanobis* distance (leverage) and the spectral residual to detect outliers in the raw spectra datasets.

Once after the outliers have been detected and identified by any kind of those mentioned methods, they should not be simply removed from the datasets, but the reasons why the outliers were present must be verified [14,15]. This can help to increase the knowledge about the data set and provide information on how to improve its quality to achieve better model performance.

### 2.5 Infrared spectra data corrections

Before performing prediction model development, it is necessary to pre-process and correct spectra data in order to achieve more accurate and robust prediction results. Several correction methods were available and can be employed based on sample characteristics, spectra impact and other related knowledge that must be recognized prior to spectra corrections. In this study, we employ three spectra correction methods namely baseline shift correction (BSC), standard normal variate (SNV) and de-trending (DT). Those three spectra corrections were then compared and see the impact on the prediction performance on nutritive parameters prediction. In NIRS practices, spectra corrections sometimes can be combined to generate a better prediction model.

### 2.6 Prediction models

The important core of NIRS practices and applications is to construct and develop models used to predict desired nutritive or quality attributes of studied samples. These quality attributes can be predicted rapidly and simultaneously through a process called as calibration, by regressing NIR spectra data (X variables) and actual measured nutritive attributes (Y variables). Ideally, the sample set employed in the regression stage must be representative of the present and of future prediction samples. It means that all expected sources of variability must be considered in both, the calibration and validation sample datasets.

In most common NIRS practices, partial least squares regression (PLSR) is one of the most widely used as regression method in constructing prediction models. The PLSR method continue to be the workhorse for regression in NIRS applications. The original PLSR is a linear method which assuming a linear relationship of the modelled nutritive parameters or concentrations as a function of the infrared spectral variations. Weak nonlinearities may be solved by increasing the number of latent variables (LVs) included in the PLSR model [12,16]. Another user preference for creating and developing prediction models in NIRS is principal component regression (PCR). It is a similar multivariate regression method works based on PCA and multiple linear regression (MLR).

The prediction performances were evaluated by means of these following statistical parameters: the coefficient of correlation (r) and determination (R^2^) between predicted and measured nutritive parameters or quality attributes, prediction error which is defined as the root mean square error (RMSE) and the residual predictive deviation (RPD), defined as the ratio between standard deviation (SD) of the population's actual value of IVOMD, IVDMD, NDF and ADF, and the RMSE of predicted nutritive parameters [1,6]. Based on literatures, good model in NIRS should have coefficient of r and R^2^ above 0.75 and RPD index above 2.5 respectively [4,17,18]. The higher value of RPD, the greater probability of models to predict desired nutritive parameters or chemical concentrations of samples dataset accurately and robustly [11,19].
